# The Effects of Shiga Toxin 1, 2 and Their Subunits on Cytokine and Chemokine Expression by Human Macrophage-Like THP-1 Cells

**DOI:** 10.3390/toxins7104054

**Published:** 2015-10-09

**Authors:** Jeremy R. Brandelli, Thomas P. Griener, Austin Laing, George Mulvey, Glen D. Armstrong

**Affiliations:** Department of Microbiology, Immunology, and Infectious Diseases, Cumming School of Medicine, University of Calgary, Calgary, AB T2N 1N4, Canada; E-Mails: jrbrande@ucalgary.ca (J.R.B.); tpgriene@ucalgary.ca (T.P.G.); aglaing@ucalgary.ca (A.L.); gmulvey@ucalgary.ca (G.M.)

**Keywords:** Shiga toxin, Stx, THP-1, Stx subunits, cytokine, HUS, STEC

## Abstract

Infection by Shiga toxin (Stx)-producing enterohemorrhagic *Escherichia coli* (EHEC) results in severe diarrhea, hemorrhagic colitis, and, occasionally, hemolytic-uremic syndrome (HUS). HUS is associated with an increase in pro-inflammatory cytokines and chemokines, many of which are produced by macrophages in the kidneys, indicating that localized host innate immunity likely plays a role in renal pathogenesis. EHEC serotypes may express one or two classes of serologically defined but structurally and functionally-related Shiga toxins called Stx1 and Stx2. Of these, Stx2 appears to be linked to higher rates of HUS than Stx1. To investigate a possible reason for this, we exposed human macrophage-like THP-1 cells to Stx1 or Stx2 and then used the Luminex multiplex system to assess cytokine/chemokine concentrations in culture supernatant solutions. This analysis revealed that, relative to Stx1, Stx2 significantly caused increased expression of GRO, G-CSF, IL-1β, IL-8 and TNFα in macrophage-like THP-1 cells. This was determined to not be due to a difference in cytotoxicity since both Stx1 and Stx2 displayed similar cytotoxic activities on macrophage-like THP-1 cells. These observations indicate that, *in vitro*, Stx2 can provoke a greater pro-inflammatory response than Stx1 in macrophages and provides a possible partial explanation for higher rates of HUS in patients infected with EHEC strains expressing Stx2. To begin to determine a mechanism for Shiga toxin-mediated cytokine production, we exposed macrophage-like THP-1 cells to Stx1 or Stx2 A and B subunits. Luminex analysis of cytokines in cell culture supernatant solutions demonstrated that neither subunit alone induced a cytokine response in THP-1 cells.

## 1. Introduction

Enterohemorrhagic *Escherichia coli* (EHEC) infection can result in severe diarrhea, hemorrhagic colitis, hemolytic-uremic syndrome (HUS) or damage to the central nervous system [1]. The most dominant serotype of EHEC causing infections worldwide is *E. coli* O157:H7 [2]. Depending on patient age and strain-related features, HUS can occur in 10% to 20% of those afflicted with EHEC-mediated hemorrhagic colitis, with children and the elderly being the more likely victims [3]. HUS is characterized by thrombocytopenia, hemolytic anemia and acute renal failure. When it occurs, HUS is believed to be initiated by Shiga toxin (Stx)-associated damage to glomerular endothelial cells which subsequently initiates an escalating cascade of localized inflammatory events that eventually lead to the clinical signs. Shiga toxins are bacterial exotoxins produced by *Shigella dysenteriae* serotype 1 and some EHEC strains. Two functionally related, yet serologically distinct Shiga toxins, Shiga toxin 1 (Stx1) and Shiga toxin 2 (Stx2), exist [4] with Stx2 being more closely associated with higher rates of HUS than Stx1 [5].

Shiga toxins are AB_5_ holotoxins composed of a single, catalytically active A subunit non-covalently associated with 5 B subunits [6]. The B subunits bind the glycolipid globotriaosylceramide (Gb_3_) receptor on host cell membranes [7] thereby initiating their entry into the cell via endocytosis resulting in their retrograde transport, first entering the trans-Golgi network then the endoplasmic reticulum (ER) [8]. While in the ER, the A subunit is proteolytically processed and activated before entering the cytosol [9] where it cleaves the 28S rRNA component of the 60S ribosomal subunit, halting peptide elongation and inhibiting protein biosynthesis in the affected cell [10]. Depurination of the 28S rRNA component results in apoptosis or activation of the ribotoxic stress response involving activation of mitogen-activated protein kinases such as p38 MAPK and results in increased expression of pro-inflammatory cytokines and chemokines [11].

Shiga toxins are necessary but not sufficient for the development of severe symptoms associated with EHEC infections. Other stimuli, including lipopolysaccharide (LPS), tumor necrosis factor α (TNFα), or interleukin-1β (IL-1β), are also required [1]. There is also clear evidence suggesting that the host innate immune response plays a role in HUS pathogenesis, with HUS patients displaying a unique cytokine profile [1] characterized by increased expression of IL-1α, IL-8, IL-10, IL-6, IL-1β and TNFα [12,13]. TNFα and IL-1β have been shown to increase the number of Gb_3_ receptors expressed on human umbilical vein endothelial cells, resulting in increased susceptibility to Shiga toxins [14]. HUS patients also show increased levels of monocyte chemotactic protein-1 (MCP-1), IL-8, macrophage inflammatory protein-1β (Mip-1β) and granulocyte colony stimulating factor (G-CSF) [15]. These chemokines could account for the increase in neutrophil and macrophage accumulation and active participation in the pathological events in the kidneys of HUS patients [16].

Previous studies have investigated the effects of Stx1 on cytokine production in phorbol 12-myristate 13-acetate (PMA) differentiated macrophage-like THP-1 cells [17,18]. However, no studies have simultaneously compared the effects of Stx1 and Stx2 on cytokine production in macrophage-like THP-1 cells. Since there is a positive correlation between Stx2 expression by EHEC and a greater risk of HUS developing as a consequence of infection, we postulated that there may be risk-related signals in the cytokine/chemokine response profile of macrophages exposed to Stx1 or Stx2. To test this hypothesis, we used the Luminex multiplex system to monitor the effects of Stx1 and Stx2 and their subunits on cytokine/chemokine production in macrophage-like THP-1 cells.

## 2. Results

### 2.1. Effects of Stx1 and Stx2 Exposure on Cytokine/Chemokine Expression by Macrophage-Like THP-1 Cells

**Figure 1:**
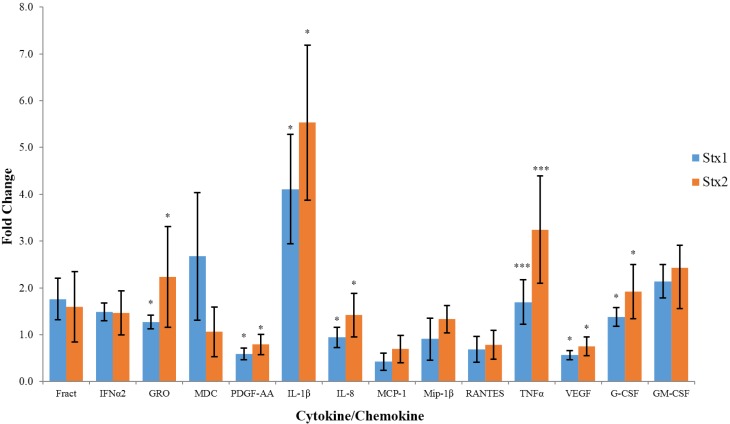
Fold change in cytokine/chemokine expression by macrophage-like THP-1. Cells were exposed to 100 ng/mL of Stx1 or Stx2 for 6 hours and cell culture supernatants were collected. Cytokine/chemokine concentrations were then determined using the Luminex multiplex assay and are presented as fold change relative to untreated cells. Data are expressed as the mean ± standard deviation for six independent trials, each performed in duplicate. Asterisks indicate significant differences (* *p* < 0.05, *** *p* < 0.001, one-way analysis of variance (ANOVA) with Tukey’s honest significant difference (HSD) test) between fold change in Stx2-, relative to Stx1-treated cells.

The data presented in Figure 1 indicate the fold change in cytokine/chemokine expression by macrophage-like THP-1 cells exposed to Stx1 or Stx2. In this case, we only present those cytokine/chemokines whose concentrations exceeded 100 pg/mL and displayed a greater than 25% change relative to their baseline concentrations detected in untreated control cells and, where Stx1 or Stx2 treatment resulted in altered levels that were significantly different from untreated cells. By these inclusion criteria, in six independent experiments, each performed in duplicate, Stx2 consistently altered concentrations of 14 out of the 40 cytokines/chemokines assessed (Table 1) in macrophage-like THP-1 cells. Of these, two (TNFα and IL-1β) are pro-inflammatory, two (G-CSF and GM-CSF) promote immune cell proliferation, four (Fractalkine, IL-8, MDC, and GRO) are chemokines, and two (PDGF-AA and VEGF) are growth-promoting factors. Only one anti-inflammatory cytokine (IFNα2) of the five (IL-1ra, IL-4, IL-10, IL-13, IFNα2) anti-inflammatory cytokines assayed was observed to be expressed at elevated concentrations by both Stx1 and Stx2-treated cells. Exposure to Stx2 also resulted in significantly higher expression of GRO, PDGF-AA, IL-1β, IL-8, TNFα, VEGF, and G-CSF relative to exposure to Stx1. Among these cytokines, IL-1β demonstrated the greatest (5.5-fold) increase upon Stx2 treatment.

The expression of GRO, IL-8, and G-CSF was significantly (*p* < 0.05) upregulated in Stx2-, relative to Stx1-treated macrophage-like THP-1 cells. IL-1β and TNFα expression was also significantly (*p* < 0.01) upregulated more so in Stx2-, relative to Stx1-treated cells while PDGF-AA and VEGF expression was significantly (*p* < 0.05) less down-regulated in Stx2-, relative to Stx1-treated cells. The expression of macrophage-derived chemokine (MDC), a chemokine for lymphocytes, monocytes, and natural killer cells, was upregulated by Stx1 relative to Stx2, which had minimal effect on its expression. However, this difference did not reach significance (*p* = 0.054).

**Table 1:** Analytes assayed in macrophage-like THP-1 cells exposed to 100 ng/mL Stx1 and Stx2.

Analytes						
EGF	Eotaxin-1	FGF-2	Flt-3L	**Fractalkine ^1^**	**G-CSF**	**GM-CSF**
**GRO**	**IFNα2**	IFNγ	IL-1α	**IL-1β**	IL-1rα	IL-2
IL-3	IL-4	IL-5	IL-6	IL-7	**IL-8**	IL-9
IL-10	IL-12(p40)	IL-12(p70)	IL-13	IL-15	IL-17A	IP-10
**MCP-1**	MCP-3	**MDC**	**Mip-1β**	**PDGF-AA**	PDGF-AB/BB	**RANTES**
sCD40L	TGF-α	**TNFα**	TNFβ	**VEGF-A**		

^1^ Analytes in bold-face type whose concentrations exceeded 100 pg/mL and displayed a greater than 25% change relative to their baseline concentrations detected in the untreated control cells and, where Stx1 or Stx2 resulted in a concentration change that was significantly different from untreated cells.

**Figure 2:**
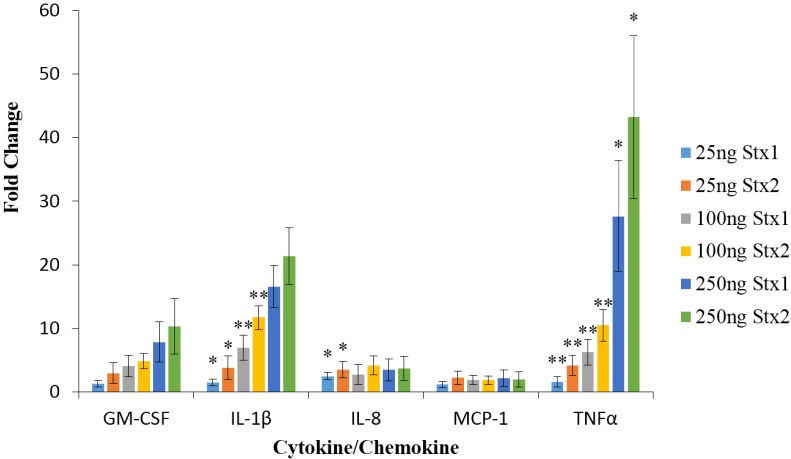
Fold change in cytokine/chemokine expression by macrophage-like THP-1 cells. Cells were exposed to 25 ng/mL, 100 ng/mL, or 250 ng/mL of Stx1 or Stx2 for 6 h and cell culture supernatants were collected. Cytokine/chemokine concentrations were then determined using the Luminex multiplex assay and are presented as fold change relative to untreated cells. Data are expressed as the mean ± standard deviation for three independent trials, each performed in duplicate. Asterisks indicate significant differences (* *p* < 0.05, ** *p* < 0.01, one-way ANOVA with Tukey’s HSD test) between fold change in Stx2-, relative to Stx1-treated cells at the same concentration.

The data in Figure 2 demonstrate that Stx2 was significantly more potent, relative to Stx1, at inducing TNFα at concentrations of 25, 100, and 250 ng/mL. At 25 and 100 ng/mL Stx2 was significantly (*p* < 0.05) more potent than Stx1 at inducing the production of IL-1β in macrophage-like THP-1 cells. At 250 ng/mL the difference between the ability of Stx2 and Stx1 to induce IL-1β production was not significant (*p* = 0.06). However, the concentration of IL-1β in the culture supernatant solutions was approaching the upper linear limit (~5000 pg/mL) for IL-1β detection by the Luminex assay in both the Stx1- and Stx2-treated macrophage-like THP-1 cell preparations. Stx2 was a more potent inducer of IL-8 at 25 ng/mL but not at 100 or 250 ng/mL. However, the concentrations of IL-8 in cells treated with 100 or 250 ng/mL of Stx1 or Stx2 were approaching the upper limit of linear detection. The analytes assayed for are listed in Table 2, only those meeting the inclusion criteria are presented in Figure 2.

**Table 2:** Analytes assayed for in macrophage-like THP-1 cells.

Analytes					
**GM-CSF ^1^**	IFNγ	**IL-1β**	IL-2	IL-4	IL-6
**IL-8**	IL-10	IL-12(p70)	**MCP-1**	**TNFα**	

^1^ Analytes in bold-face type whose concentrations exceeded 100 pg/mL and displayed a greater than 25% change relative to their baseline concentrations detected in the untreated control cells and, where Stx1 or Stx2 resulted in a concentration change that was significantly different from untreated cells.

### 2.2. Effects of Stx1 and Stx2 Exposure on the Viability of Macrophage-Like THP-1 Cells

**Figure 3:**
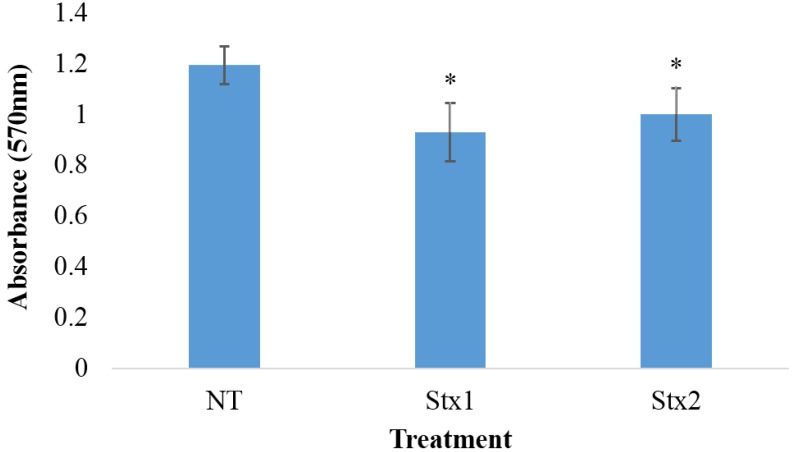
The viability of macrophage-like THP-1 cells exposed to Stx1 or Stx2. Macrophage-like THP-1 cells were exposed to 100 ng/mL of Stx1 or Stx2 or left untreated (NT) for 6 hours. An MTT assay was then performed and the absorbance of viable cells was determined at 570 nm. The data represent the means of three trials performed in triplicate ± standard deviation. Asterisks indicate significant differences between Stx treated and untreated (NT) cells (* *p* < 0.01, one-way ANOVA with Tukey’s HSD test) between treated and untreated cells.

The cell viability data presented in Figure 3, as assessed by the AD(P)H-dependent cellular oxidoreductase-based MTT assay, indicate that, although the viability of macrophage-like THP-1 cells exposed to 100 ng/mL of Stx1 or Stx2 for 6 h decreased by 17% to 20% relative to untreated cells, no significant difference in cell viability was observed between the Stx1 and Stx2 treated cells.

### 2.3. Effects of StxA and StxB Subunits on Cytokine/Chemokine Expression by Macrophage-Like THP-1 Cells

**Figure 4:**
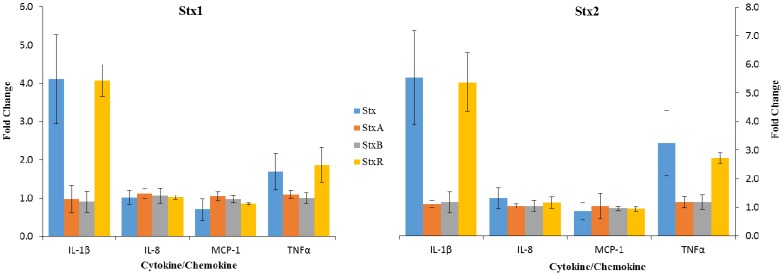
The effects of Stx1, Stx2 and their A and B subunits on cytokine production in macrophage-like THP-1 cells. Macrophage-like THP-1 cells were incubated with 100 ng/mL of Stx1 or Stx2 or recombined Stx1 (Stx1R) or Stx2 (Stx2R) or were incubated with equimolar concentrations of their A or B subunits (3.125 nmol or 15.625 nmol, respectively). The recombined toxins were produced by incubating 5 mol of B with 1 mol of A subunit on ice overnight. Cell culture supernatant solutions were then collected and analyzed for cytokine/chemokine concentrations using Luminex multiplex analysis. The values represent the means of three independent trials performed in duplicate ± standard deviation for Stx1 and Stx2, or their A and B subunits. The assay was only performed once in duplicate for Stx1R and Stx2R.

Shiga toxin subunits were isolated and assayed for purity and Verocytotoxic activity before being used to treat macrophage-like THP-1 cells (Figures S1–S6). The data presented in Figure 4 demonstrate the effects of equimolar concentrations of isolated Stx1 and Stx2 A and B subunits on cytokine and chemokine expression by macrophage-like THP-1 cells. Only those cytokine/chemokines whose concentrations exceeded 100 pg/mL are presented. None of 11 cytokines assayed (Table 2), displayed significantly elevated concentrations in the Stx1 and Stx2 A or B subunit-treated, relative to untreated, cells. The recombined Shiga toxins (StxR) displayed no significant difference in activity from the holotoxin for all of the cytokines meeting the inclusion criteria (Table 2). The activity of Stx1 was significantly different from that of both Stx1A and Stx1B for cytokines meeting the inclusion criteria with the exception of IL-8, with none of the treatments resulting in altered IL-8 levels relative to untreated cells. The activity of Stx2 was significantly different from that of both Stx2A and Stx2B for the cytokines meeting the inclusion criteria with the exception of MCP-1, with none of the treatments resulting in altered MCP-1 levels relative to untreated cells.

## 3. Discussion

Renal biopsy specimens obtained from patients suffering from HUS reveal increased numbers of macrophages and monocytes [19]. This coincides with an increase in urinary IL-8 concentrations, indicating a role for IL-8 in recruiting macrophages, neutrophils and monocytes to the kidneys of HUS patients [19]. Macrophages have also been shown to be responsible for glomerular fibrin deposition [20]. Fibrin-rich thrombi in renal microvasculature are observed in HUS patients and apparently play a role in decreasing renal function [16,21]. The results presented in Figure 1 and Figure 2 reveal that, *in vitro*, Stx2 is able to cause a significantly higher increase in IL-8 production in macrophage-like THP-1 cells relative to Stx1. Furthermore, the data in Figure 1 shows that Stx1 was unable to produce a change in IL-8 concentrations that were significantly different from untreated cells (One sided *t*-test, *p* = 0.7162). Stx2 is also able to up-regulate G-CSF expression in macrophage-like THP-1 cells, which could explain the increased population of monocytes and macrophages present in the kidneys of HUS patients. These observations indicate that Stx2, more so than Stx1, may play a role in macrophage infiltration and fibrin deposition in a cytokine-mediated manner in HUS patients.

TNFα and IL-1β may also contribute to the vascular damage observed in EHEC-infected individuals. It was previously reported that exposing human umbilical vein endothelial cells to these cytokines induces increased Gb_3_ expression, therefore facilitating increased Stx1 and Stx2 binding [14]. IL-1β and TNFα displayed the largest fold increase out of all of the cytokines assayed at every concentration of toxin used. Moreover, TNFα and IL-1β have also both been observed at increased concentrations in the urine and sera of children suffering from HUS [21]. TNFα and IL-1β may play an important role in the pathogenic cascade of HUS. The data in this study indicate that Stx2 is a more potent inducer of both cytokines. This may provide evidence that Stx2 is more closely linked to HUS through cytokine induction.

The effects of Stx1 and Stx2 on cell viability were also determined to ensure that differences seen between the toxins were due to a difference in pro-inflammatory activity and not differences in overall cytotoxicity. Stx1 and Stx2 displayed similar toxicity in Vero cells and macrophage-like THP-1 cells, as determined by the Verocytotoxicity and MTT assays, respectively. This indicates that both toxins have similar cytotoxic activity and the difference in cytokine induction isn’t the result of a difference in cytotoxic potency or purity of the toxin preparations. Moreover, although the difference did not reach significance Stx1 consistently caused up-regulation of MDC relative to Stx2 (Figure 1), further demonstrating that the differential effects of Stx1 and Stx2 on cytokine induction was not simply dose-related.

The mechanism by which the Shiga toxins induce cytokine expression in macrophage-like THP-1 cells has not yet been fully revealed. By exposing macrophage-like THP-1 cells to isolated Shiga toxin A and B subunits we sought to determine if the interaction of the toxin subunits with their receptors was sufficient to elicit a cytokine response. The results presented in Figure 4 showed that neither the A nor B subunits of Stx1 or Stx2 induce a cytokine response. Further, the observation that the Shiga toxin A and B subunits were able to recombine into active holotoxins (Figure 4) revealed that they were likely folded into their native conformations. Their failure to produce an elevated cytokine response in macrophage-like THP-1 cells was, therefore, not the result of unfolding. These results suggest that, either subunit alone engaging a receptor on macrophage-like THP-1 cells does not result in increased cytokine production or, that absent their B subunits, the Shiga toxin A subunits cannot interact with the cells in a manner that results in uptake or intracellular activation. These data provide support for the hypothesis that the Shiga toxins induce increased cytokine production through activation of the ribotoxic stress response, which is consistent with the findings of previous studies [18,22]. Further, the JNK and p38 pathways implicated in upregulating cytokine production upon exposure to Shiga toxin are not activated through engagement of a receptor alone.

It is well documented that human serum amyloid P component (HuSAP), a member of the pentraxin superfamily of conserved acute phase proteins, binds to and neutralizes the cytotoxic activity of Stx2 but not Stx1 *in vitro* [23] and also in HuSAP-expressing transgenic mice receiving an injection of purified LPS in addition to Stx2 [24]. Accordingly, it is possible that, while the results reported herein indicate that Stx1 is less pro-inflammatory than Stx2, these experiments were conducted in an environment absent HuSAP which could influence the activity of these toxins in EHEC-infected subjects. However, we also previously reported that HuSAP does not inhibit Gb_3_-independent Stx2 binding to human neutrophils, influence their expression of cytokines upon exposure to Stx2 *in vitro*, nor cytokine gene expression in the kidneys of Stx2-susceptable LPS-treated HuSAP-transgenic mice [24].

## 4. Experimental Section

### 4.1. THP-1 Cell Culture and Differentiation

The human myelogenous leukemia cell line THP-1 [25] was acquired from the American Type Culture Collection (Rockville, MD, USA). The cells were cultured as instructed at 37 °C and 5% CO_2_ in a humidified incubator in Roswell Park Memorial Institute (RPMI) 1640 medium (Gibco, Grand Island, NY, USA) supplemented with 10% heat-inactivated fetal bovine serum, 0.05 mmol/L β-mercaptoethanol, and 1% sodium pyruvate. THP-1 cells were differentiated into macrophage-like cells by exposing 1 × 10^6^ cells/mL in a 24-well tissue culture plate to 100 ng/mL phorbol 12-myristate 13-acetate (PMA) for 48 h. Adherent cells were washed twice with serum-free Opti-MEM medium to remove non-differentiated cells and PMA. Cells were then cultured for 1 day in PMA-free culture medium prior to use.

### 4.2. Shiga Toxins

Stx1 and Stx2 were affinity purified using Synsorb-Pk as previously reported [26]. Residual LPS was removed using Endotrap blue LPS affinity columns [26] with the final concentration of LPS determined to be <0.001 endotoxin units per μg of toxin as assessed by the colorimetric Limulus amebocyte lysate assay. Toxins were diluted to 10 μg/mL in phosphate-buffered (pH 7.2) physiological saline (PBS) and stored at −80 °C prior to use. The purity of the toxin preparations was assessed by SDS polyacrylamide gel electrophoresis and a Verocytotoxicity assay followed by a Giemsa stain [27]. Briefly, the Verocytotoxicity assay was performed by seeding a 96-well plate with Vero cells and left to rest overnight. Media was then changed and cells were exposed to serial dilutions of toxin for 48h before being Giemsa stained. Briefly, media was removed, cells were methanol fixed, exposed to Giemsa stain and the absorbance at 620 nm was recorded. The assays were performed to ensure that the Stx1 and Stx2 preparations displayed similar relative specific activities. The results of these assays are presented as supplemental Figures S7 and S8, respectively.

### 4.3. Macrophage-Like THP-1 Cell Cytokine/Chemokine Analysis

Differentiated THP-1 cells were incubated in a minimum amount of growth medium in the presence of Stx1 or Stx2 for 6 h. The culture medium was then collected and centrifuged at 1000× *g* for 10 min to remove cells and cell debris and the resulting supernatant solutions were retained for analysis. Samples were analyzed for cytokine/chemokine concentrations using the 40-Plex (Table 1) Primary Cytokine/Chemokine Panel Luminex Assay (Eve Technologies, Calgary, AB, Canada).

### 4.4. MTT Assay

THP-1 cells were cultivated at 2 × 10^5^ cells/well in 96-well tissue culture plates and exposed to 100 ng/mL of PMA for 48 h. Adherent cells were washed twice with serum-free Opti-MEM medium to remove non-differentiated cells and PMA. The cells were incubated for 1 day in PMA-free culture media and then incubated in 200 µL of serum-free medium and exposed to Stx1 or Stx2 at 100 ng/mL for 6 h. The MTT assay (TREVIGEN 4890-25-K) was performed as outlined in the manufacturer’s manual. Briefly, 10 µL of MTT reagent was added to each well and incubated for 4 h. 100 µL of detergent reagent was added and left to incubate overnight before recording the absorbance in each well at 570 nm using a microplate reader.

### 4.5. Shiga Toxin Subunit Separation and Evaluation

Preparation of the Shiga toxin subunits involved separating them from the intact holotoxins using high-performance liquid chromatography (HPLC, GE Life Sciences, Mississauga, ON, Canada) as previously described [28]. Briefly, Stx1 and Stx2 were incubated in a dissociating solution (9 M urea, 0.15 M NaCl, 0.15 M propionic acid, pH 4.0) for 1 h on ice. The subunits were then separated by HPLC size exclusion gel filtration chromatography (Superdex 75 GE Life Sciences 17-5174-01, Mississauga, ON, Canada). The separation (Figures S1 and S2) was accomplished at 0.60 mL/min with 1.5 column volumes of ice-chilled 6 M urea, 0.15 M NaCl, 0.15 M propionic acid (pH 4.0) solution. The fractions were subsequently concentrated using 10,000 molecular weight cut-off ultra-centrifugal filters (Millipore RK-299638-30, Billerica, MA, USA) and re-suspended in PBS. The Shiga toxin subunits were subsequently assayed for protein concentration using the bicinchoninic acid assay (BCA). Each subunit was analyzed for purity by SDS polyacrylamide gel electrophoresis (Figures S3 and S4) and for cytotoxicity using the Verocytotoxicity assay followed by a Giemsa stain (Figures S5 and S6) as previously described [27]. The reassembled Shiga toxins were produced by mixing 5 mol of B subunit with 1 mol of A subunit and incubated on ice overnight to allow the subunits to reassemble into holotoxins.

### 4.6. Macrophage-Like THP-1 Cell Cytokine/Chemokine Analysis for the Effects of Shiga Toxin A and B Subunits

PMA-differentiated macrophage-like THP-1 cells were exposed to 3.125 nmol of the Shiga toxin A subunit or 15.625 nmol of the Shiga toxin B subunit for 6 h. Cell culture supernatant solutions were then collected and analyzed for cytokine/chemokine concentrations using the Cytokine/Chemokine Panel Luminex Assay.

### 4.7. Data Analysis

Cytokine/chemokine concentrations recorded in Stx-treated THP-1 cell supernatant solutions were normalized relative to those detected in untreated solutions and expressed as the fold change in concentration. Statistical evaluation of differences in cytokine/chemokine concentrations was performed using Stata (StataCorp LP, College Station, TX, USA) employing one-way ANOVA with Tukey’s honest significance difference (HSD) or by the Student’s *t*-test assuming unequal variance. A *p* value < 0.05 was considered to indicate a significant difference. All data presented represent the mean and standard deviations for the indicated number of independent experiments each performed in duplicate.

## 5. Conclusions

Taken together, the data presented herein demonstrate that, *in vitro* at least, Stx2 can induce macrophage-like THP-1 cells to express greater amounts of pro-inflammatory cytokines and chemokines than Stx1. The difference in pro-inflammatory activity doesn’t appear to be linked to the cytotoxic activity of the toxins or the ability of either StxA or StxB to interact with a cell. These findings are consistent with HUS pathological signs and could partially explain the greater association between EHEC strains expressing Stx1 and Stx2 or Stx2 alone and the risk of the sequela developing in EHEC-infected subjects.

## Supplementary Materials

Supplementary materials can be accessed at: http://www.mdpi.com/2072-6651/7/10/4054/s1.
