# Supplementary material for: The Effects of Shiga Toxin 1, 2 and Their Subunits on Cytokine and Chemokine Expression by Human Macrophage-Like THP-1 Cells
Source: Toxins (Basel). 2015 Oct 9;7(10):4054–66. doi: 10.3390/toxins7104054 (PMC4626720; doi:10.3390/toxins7104054)
Supplement: Supplementary file 1 [file toxins-07-04054-s001.pdf]

Supplementary Materials

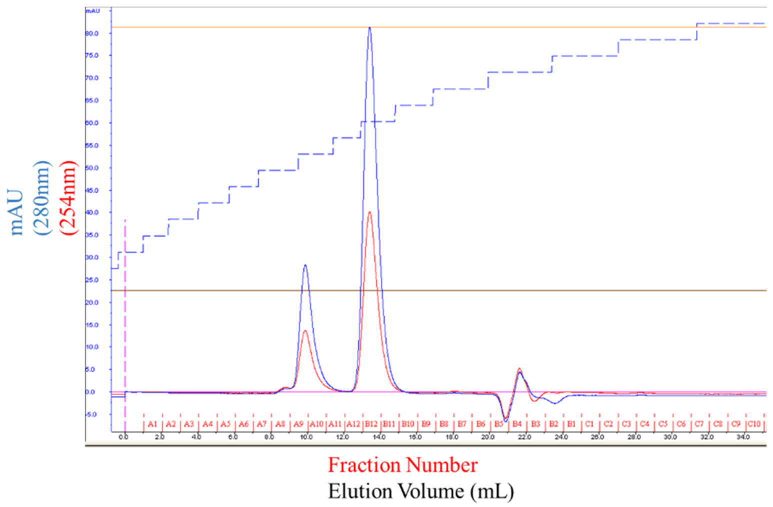

Figure S1. Absorbance measurements from the HPLC separation of Stx1 into Stx1A and Stx1B. The red lines represent absorbance at 254 nm and the blue lines represent absorbance at 280 nm as measured in absorbance units on the y-axis (mAU). The x-axis represents the tube numbers and elution volume. 2.0 mg of Stx1 was incubated in 9 M urea, 0.15 M NaCl, 0.15 M propionic acid, pH 4.0 for 1 hour on ice. The Stx1 was then loaded into the HPLC machine using a running buffer composed of 6 M urea, 0.15 M NaCl, 0.15 M propionic acid, pH 4.0 kept at 4 °C. The flow rate was 0.6 mL/min. Fractions A8-A10 and A12-B11 were collected for Stx1A and Stx1B, respectively. These samples were then concentrated and resuspended in PBS.

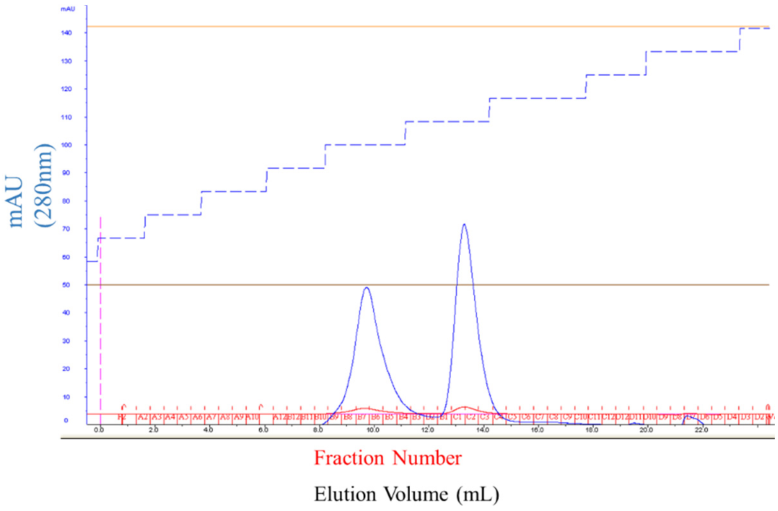

Figure S2. Absorbance measurements from the HPLC separation of Stx2 into Stx2A and Stx2B. The blue lines represent absorbance at 280 nm as measured in absorbance units on the y-axis (mAU). The *x*-axis represents the tube numbers and elution volume. 2.0 mg of Stx2 was incubated in 9 M urea, 0.15 M NaCl, 0.15 M propionic acid, pH 4.0 for 1 h on ice. The Stx2 was then loaded into the HPLC machine using a running buffer composed of 6 M urea, 0.15 M NaCl, 0.15 M propionic acid, pH 4.0 kept at 4 °C. The flow rate was 0.6 mL/min. Fractions B10-B4 and B1-C4 were collected for Stx2A and Stx2B, respectively. These samples were then concentrated and resuspended in PBS.

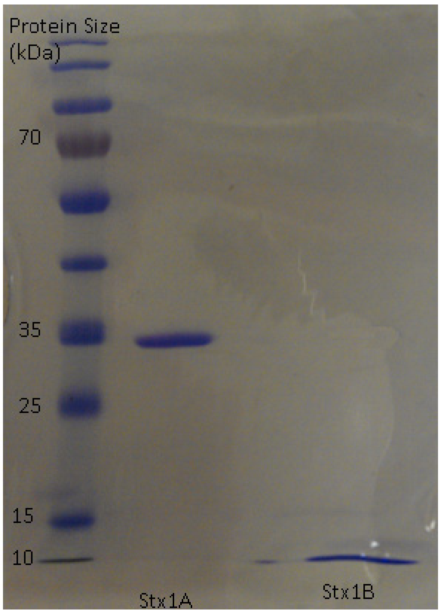

Figure S3. Polyacrylamide gel electrophoresis of Stx1A and Stx1B. PAGE was run using 15 µL of each sample (250 ng) at 100 V for 2 h. The gel was then stained with Coomassie blue, destained and imaged. ThermoScientific PageRuler Prestained Protein ladder was used (Product #26616) to calibrate the gel. Stx1A and Stx1B represent samples obtained from incubating 2.0 mg of Stx1 with 9 M urea, 0.15 M NaCl, 0.15 M propinic acid, pH 4.0 on ice for 1 h then separating the subunits using HPLC. The band at ~32 kDa represents the Stx1A subunit and the band parallel with 10 kDa represents the Stx1B subunit.

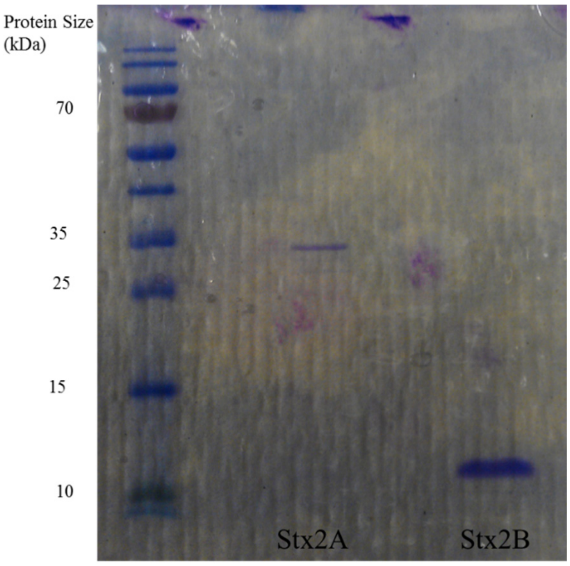

Figure S4. Polyacrylamide gel electrophoresis of Stx2A and Stx2B. PAGE was run using 15 µL of each sample (250 ng) at 100 V for 2 h. The gel was then stained with Coomassie blue, destained and imaged. ThermoScientific PageRuler Prestained Protein ladder was used (Product #26616) to calibrate the gel. Stx2A and Stx2B represent samples obtained from incubating 2.0 mg of Stx2 with 9 M urea, 0.15 M NaCl, 0.15 M propinic acid, pH 4.0 on ice for 1 h then separating the subunits using HPLC. The band at ~32 kDa represents the Stx2A subunit and the band parallel with 10 kDa represents the Stx2B subunit.

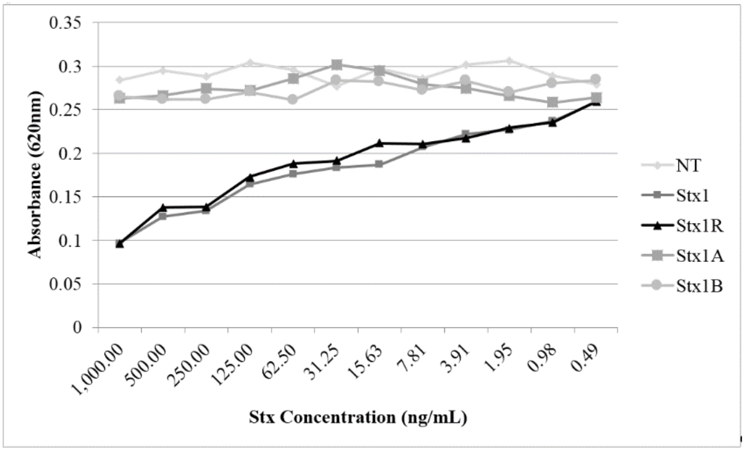

Figure S5. Verocytotoxicity analysis of Stx1 and its subunits. Vero cells (ATCC CCL-81) were seeded in a 96-well plate with MEM + 10% FBS overnight until cell layers were confluent. Cells were then exposed to Stx1, Stx1A subunit, Stx1B subunit or reconstituted Stx1 (Stx1R), serially diluted 2 fold across the plate. The plate was then incubated for 48 h at 37 °C, 5% CO_2. Medium was removed, cells were fixed with methanol and Giemsa stained. After the plate had dried the absorbance at 620 nm was recorded, providing the optical density of the cells, a measure of cell viability. Optical density values were plotted against the concentration of Shiga toxin present in the wells. Stx1R was created by creating a 5 M Stx1B and 1 M Stx1A solution and leaving it on ice overnight to allow the subunits to recombine. The data graphed represent the mean of one trial performed in triplicate.

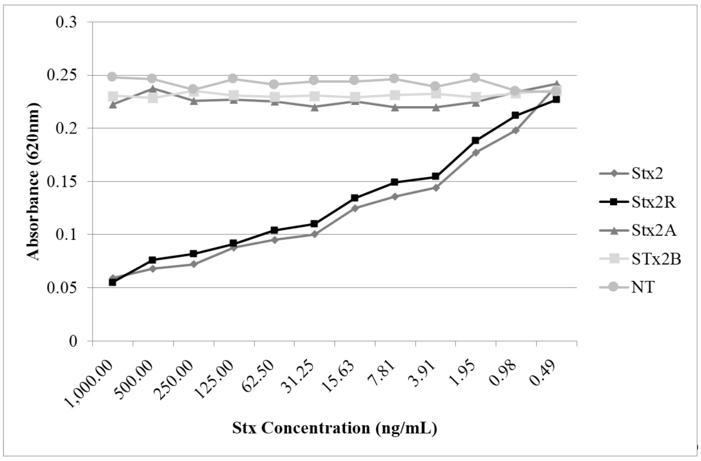

Figure S6. Verocytotoxicity analysis of Stx2 and its subunits. Vero cells (ATCC CCL-81) were seeded in a 96-well plate with MEM + 10% FBS overnight until cell layers were confluent. Cells were then exposed to Stx2, Stx2A subunit, Stx2B subunit or reconstituted Stx2 (Stx2R), serially diluted 2 fold across the plate. The plate was then incubated for 48 h at 37 °C, 5% CO_2. Medium was removed, cells were fixed with methanol and Giemsa stained. After the plate had dried the absorbance at 620 nm was recorded, providing the optical density of the cells, a measure of cell viability. Optical density values were plotted against the concentration of Shiga toxin present in the wells. Stx2R was created by creating a 5 M Stx2B and 1 M Stx2A solution and leaving it on ice overnight to allow the subunits to recombine. The data graphed represent the mean of one trial performed in triplicate.

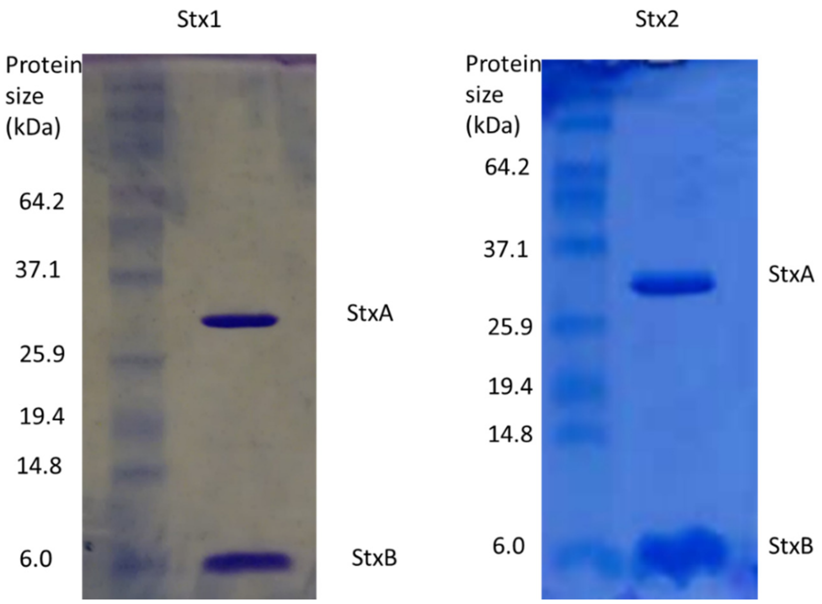

Figure S7. Sodium dodecyl sulfate polyacrylamide gel electrophoresis of Stx1 and Stx2. 2 µg of each Stx was added to loading dye mix, boiled and loaded into a 12.5% polyacrylamide gel. The gel was run at 100 V for 2 h, stained with Coomassie blue and imaged. ThermoScientific PageRuler Prestained Protein ladder was used (Product #26616) to calibrate the gel. The band between 37.1 and 25.9 kDa represents the StxA subunit and the band around 6.0 kDa represents the StxB subunit.

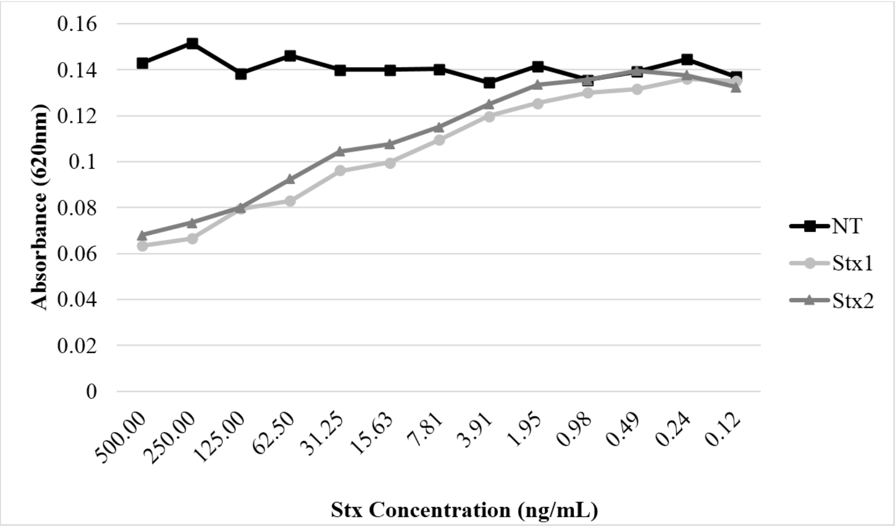

Figure S8. Verocytotoxicity assay comparing the cytotoxic activity of Stx1 and Stx2. Vero cells (ATCC CCL-81) were seeded in a 96-well plate with MEM + 10%FBS overnight until cell layers were confluent. Cells were then exposed to Stx1 or Stx2 and serially diluted 2 fold across the plate. The plate was then incubated for 48 h at 37 °C, 5% CO_2. Medium was removed, cells were fixed with methanol and Giemsa stained. After the plate had dried the absorbance at 620 nm was recorded, providing the optical density of the cells, a measure of cell viability. Optical density values were plotted against the concentration of Shiga toxin present in the wells. The data graphed represent one trial performed in triplicate for Stx1 and Stx2 and duplicate for no treatment (NT).
